# Fluorescence Quantum Yield of Thioflavin T in Rigid Isotropic Solution and Incorporated into the Amyloid Fibrils

**DOI:** 10.1371/journal.pone.0015385

**Published:** 2010-10-29

**Authors:** Anna I. Sulatskaya, Alexander A. Maskevich, Irina M. Kuznetsova, Vladimir N. Uversky, Konstantin K. Turoverov

**Affiliations:** 1 Institute of Cytology, Russian Academy of Sciences, St. Petersburg, Russia; 2 Yanka Kupala Grodno State University, Grodno, Belarus; 3 Institute of Biological Instrumentation, Russian Academy of Sciences, Pushchino, Russia; 4 Department of Biochemistry and Molecular Biology, Indiana University School of Medicine, Indianapolis, Indiana, United States of America; Griffith University, Australia

## Abstract

In this work, the fluorescence of thioflavin T (ThT) was studied in a wide range of viscosity and temperature. It was shown that ThT fluorescence quantum yield varies from 0.0001 in water at room temperature to 0.28 in rigid isotropic solution (*T/η*→0). The deviation of the fluorescence quantum yield from unity in rigid isotropic solution suggests that fluorescence quantum yield depends not only on the ultra-fast oscillation of ThT fragments relative to each other in an excited state as was suggested earlier, but also depends on the molecular configuration in the ground state. This means that the fluorescence quantum yield of the dye incorporated into amyloid fibrils must depend on its conformation, which, in turn, depends on the ThT environment. Therefore, the fluorescence quantum yield of ThT incorporated into amyloid fibrils can differ from that in the rigid isotropic solution. In particular, the fluorescence quantum yield of ThT incorporated into insulin fibrils was determined to be 0.43. Consequently, the ThT fluorescence quantum yield could be used to characterize the peculiarities of the fibrillar structure, which opens some new possibilities in the ThT use for structural characterization of the amyloid fibrils.

## Introduction

The deposition of proteins in the form of regular amyloid fibrils represents a pathological hallmark of several human diseases [1], [2], [3], [4], [5], [6], [7]. Depending on the disease, such proteinaceous deposits can be found in the brain, vital organs such as the liver and spleen, or skeletal tissue, depending on the disease [6], [7]. The protein deposition diseases are among the most costly and debilitating health disorders. Many of them, such as Alzheimer's and Parkinson's diseases and late-onset diabetes, are age-related and are becoming increasingly prevalent in the modern world. Although fibrils from different pathologies display many common morphological and structural properties, the more than 20 proteins known to be involved in deposition diseases are structurally unrelated [6], [7], [8], [9]. These amyloidogenic proteins may be well-folded proteins or intrinsically unstructured [9]. There is an increasing belief that the ability to fibrillate is a generic property of a polypeptide chain, and that all proteins are potentially able to form amyloid fibrils under appropriate conditions [8], [9], [10], [11], [12]. It has been established that protein aggregation involves a unifying mechanism where the structural transformation of a polypeptide chain into a partially folded or misfolded conformation represents a first crucial step [8], [9]. Therefore, understanding the nature and structural features of different partially folded and misfolded conformations represents a crucial step in fundamental science, biotechnology and medicine.

**Figure 1:**
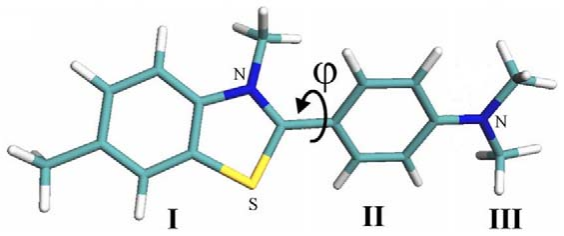
Model of thioflavin T molecule.

Thioflavin T (ThT, Figure 1) is a common tool for diagnostics of the amyloid fibril formation [13], [14], [15], [16], [17], [18], [19], [20]. Importantly, ThT interaction with amyloid fibrils is highly specific, as this dye does not interact with proteins in their folded, unfolded or partially folded monomeric forms or at least the formation of dye-monomeric protein complexes is not accompanied by the changes in the dye spectral properties. Therefore, due to these unique properties, ThT represents a useful and convenient diagnostic tool for the fast and reliable identification of amyloid fibrils in disease-affected tissues and organs. Furthermore, in *in vitro* fibrillation studies, the appearance of the specific ThT fluorescence is considered to be an indication of the amyloid fibril formation [21], [22], [23], [24]. This approach is widely accepted and the number of studies based on ThT diagnostic capabilities is rapidly growing. The current status of ThT in the investigation of amyloid fibrils is given in a recent in-depth review [25]. Although it is of great importance for studies of amyloid fibrils, the molecular mechanisms of the specific ThT binding to these structures and the reasons underlying the characteristic increase in the ThT fluorescence quantum yield accompanying the incorporation of this dye into the fibrils are not yet fully understood. A model in which ThT incorporates into fibrils in its monomeric form [26] is in agreement with the explanation of the significant (several orders of magnitude) increase in the ThT fluorescence intensity induced by restriction of torsion oscillations of its fragments [27], [28], [29], [30]. Further support for this model came from the study of the dependence of ThT fluorescence quantum yield and lifetime of the excited state on solvent viscosity and temperature [31]. However, the model analyzed in previous studies proposed that the ThT fluorescence quantum yield should be equal to unity when oscillations of the dye's fragments relative to each other are completely restricted. In this work, a significantly greater range of temperature and viscosity were analyzed, and a new interpretation of the experimental data was given that took into account a non-planar conformation of the ThT molecule in the ground state. The use of the fluorescence dye ATTO-425 with a known quantum yield and spectral characteristics similar to that of ThT has enabled a more accurate evaluation of the absolute values of the ThT fluorescence quantum yield in solutions of different viscosity and therefore, an estimation of the value of the radiative lifetime of the excited state of the dye. It is concluded that the conformation of ThT molecule in ground state affects its fluorescence quantum yield. The validity of this model was proven by the estimation of fluorescence quantum yield of ThT incorporated into the insulin amyloid fibrils.

## Results and Discussion

### ThT fluorescence in solvents with different viscosity

**Figure 2:**
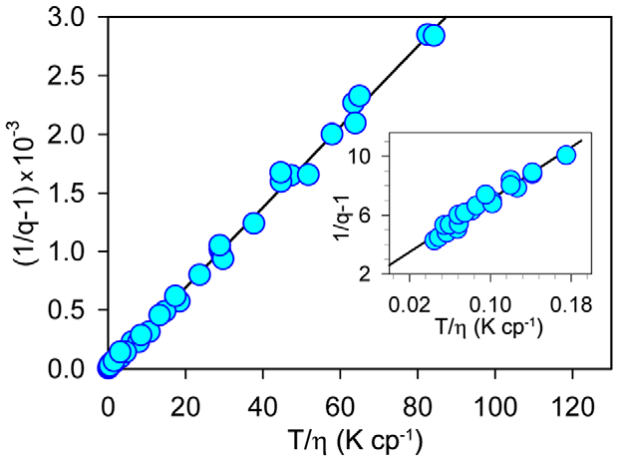
Dependence of thioflavin T fluorescence quantum yield on solvent viscosity and temperature in coordinates 1/*q* vs. *T*/*η*. Solvent viscosity was changed by variation of glycerol content from 13 to 99% in water-glycerol mixtures. Solvent temperature was changed from 3 to 50°C. **Insert**. The section of the plot corresponding to solutions of high viscosity glycerol content from 96 to 99% and low temperature from 3 to 10°C is given in the extended scale.

**Figure 3:**
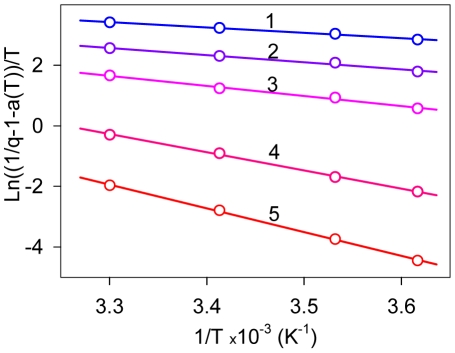
Dependence of thioflavin T fluorescence quantum yield on solvent temperature in the coordinates 
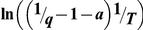
 vs. 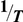 for solutions of different viscosity. Curves 1–5 correspond to 13, 35, 56, 83 and 99% wt of glycerol content, respectively.

The dependence of ThT fluorescence quantum yield and excited state lifetime on solvent viscosity and temperature was determined in water-glycerol mixtures. Glycerol content was varied from 13 to 99% by weight (wt) and solvent temperature ranged from 3 to 50°C. The experimental data in the form of 1/*q*−1 vs. *T*/*η* plot forms a straight line (Figure 2). The intercept on the ordinate axis is larger than 2.0 (Figure 2; Insert). Figure 3 represents the dependence of the ThT fluorescence quantum yield on temperature in water-glycerol mixtures with different glycerol contents in the 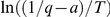 vs. 1/*T* coordinates. The choice of the coordinates for the presentation of experimental data in Figures 2 and 3 is explained below. Experimental values for fluorescence quantum yield and the excited states lifetimes of ThT in 96–99% wt glycerol at different temperatures are summarized in Table 1.

**Table 1:** Emission Quantum Yield, Excited-State Lifetime and Radiative Lifetime of Thioflavin T in Solutions with Different Glycerol Content at Different Temperatures.

glycerol content, % wt	*t*, °C	*q*	*τ*, ns	*τ*_r_, ns
99	5	0.158	1.14	7.18
	7	0.156	1.02	6.51
	10	0.125	0.91	7.29
	20	0.066	0.48	7.27
98	5	0.142	0.98	6.89
	7	0.131	0.91	6.96
	10	0.113	0.82	7.30
96	5	0.119	0.90	7.54
	7	0.106	0.77	7.28
	10	0.091	0.73	8.05
	20	0.046	0.35	7.61
0	20	∼0.0001^a^	∼0.0008^c^	–
*η*→∞	*T*→0	0.28^a^	2.2^b^	7.8

a the value was determined by extrapolation of the dependence given in Figure 2;

b the value was obtained for thioflavin T in 99% glycerol at 77 K [31].

c the value was evaluated as *τ*_r_ q, average *τ*_r_ was taken as 7.8 ns.

There are three structural fragments in the thioflavin T molecule: the benzothiazole ring (I), the benzyl rings (II) and the dimethylamino group (III) (Figure 1). Photophysical properties of this dye are substantially determined by the methyl group at N5 atom of benzothiazole ring [28], [30]. Van der Waals repulsion between this group and hydrogen atoms of the benzyl ring makes the planar conformation energetically unfavorable (Figure 1) and causes an energy barrier for torsional oscillation of benzothiazole and aminobenzoyl rings relative each other at *ϕ* = 0 (180)°. The other internal rotation barrier at *ϕ* = 90 (270)° is due to the disturbance of the uniform system of π-conjugated bonds of the benzothiazole and aminobenzoyl rings. The presence of the methyl group at N5 atom of benzothiazole ring not only prevents a planar configuration of the ThT molecule but also diminishes the energy barrier at *ϕ* = 90 (270)° between states corresponding to energy minima at *ϕ* = 37 and 145° and at *ϕ* = 217 and 325°. Different approaches evaluate this barrier as 2.0 or 3.4–4.3 kcal·mol^−1^. A ThT analog with a methyl group at the N5 atom of the benzothiazole ring replaced by a hydrogen atom has an energy minimum at *ϕ* = 0 (180)° separated by energy barriers of 11.4 kcal·mol^−1^ at *ϕ* = 90 (270)°.

Quantum-chemical calculations revealed that the isolated ThT molecule energy in the excited state is monotonously decreases with the *ϕ* increase from 0 to 90°, where it reaches its minimal value [29], [30]. Thus, the conformation with the disturbed π-conjugated bond system became energetically favorable. Calculations also showed that for the isolated ThT molecule, there was no energy barrier preventing the molecule from transfer to the state, which, caused the non-radiative deactivation of the excited state as postulated [29], [30]. For ThT molecules in water and alcohol solutions, there are at least two factors preventing the transition from the radiative to the non-radiative state with the disturbed system of π-conjugated bonds: electrostatic interaction with the solvent and solvent viscosity. The obtained experimental data (Figure 2 and Table 1) showed that solvent heating or a decrease in its viscosity (by a change in the water-glycerol content) is accompanied by a dramatic decrease in ThT fluorescence quantum yield and average fluorescence decay time. This means that there is a deactivation process of the excited state of ThT molecule, the rate constant of which depended on solvent temperature and viscosity. As previously suggested [29], [30], such a deactivating process could be torsional oscillations of the benzothiazole and aminobenzyl rings relative to each other. As the minimum of ThT molecule energy in excited state is at *ϕ* = 90 (270)°, this process will lead to the change of the initial distribution of *ϕ* angle causing an increase in the fraction of molecules with larger *ϕ*. We propose that this process, which is accompanied by a decrease in the conjugation of the π-electron system of the benzothiazole and aminobenzyl rings of ThT molecule, can lead to the increase in the non-radiative deactivation of the ThT excited state. According to the Debye-Stokes-Einstein law, the rate constant of this process will be proportional to the ration *T/η* (see e.g. [32]):
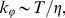
(1)where *T* is the absolute temperature and *η* is the solvent viscosity. Taking into account that even in the ground state the *ϕ* angle between benzothiazole and aminobenzyl rings does not equal to zero, we have:
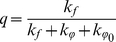
(2)and
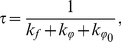
(3)where 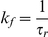 is the rate constant of the deactivation process of the excited state with radiation, 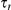 is the radiative lifetime of the excited state, 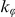 is the rate constant of the ThT molecules reaching the non-fluorescent state with *ϕ* = 90 (270)°, which usually called TICT (twisted internal charge transfer) and 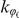 is the rate constant of the excited state deactivation when *T*→0, *η*→∞, i.e. when 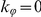. This state is known as the LE state (local excited). It is likely that deviation of the ThT fluorescence quantum yield from unity in the absence of torsional oscillations of the benzothiazole and aminobenzyl rings relative to each other is caused by the non-planar conformation of ThT molecules in Franck-Condon excited state just after excitation, with a maximum *ϕ* angle distribution close to *ϕ*_0_ = 37° [29]. We suggest that the *ϕ* angle distribution of ThT molecules incorporated in amyloid fibrils can differ from that in rigid isotropic solutions and this can be an important factor in determining the fluorescence quantum yield of bound ThT. Within the frame of the given assumption for ThT in solution, we have:
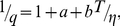
(4)where 
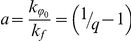
 at *T*→0 и *η*→∞. Our experimental data showed that the value 1/*q* determined for ThT in water-glycerol mixtures with different glycerol content and at different temperatures is linearly dependent on *T*/*η* (Figure 3). This provides strong support for the model of ThT non-radiative deactivation described above. A relationship similar to (7) was used to describe the ThT fluorescence quantum yield dependence on microviscosity in inverted AOT micelles with different ratios of *w_0_* = [H_2_O]/[AOT] [33].

**Figure 4:**
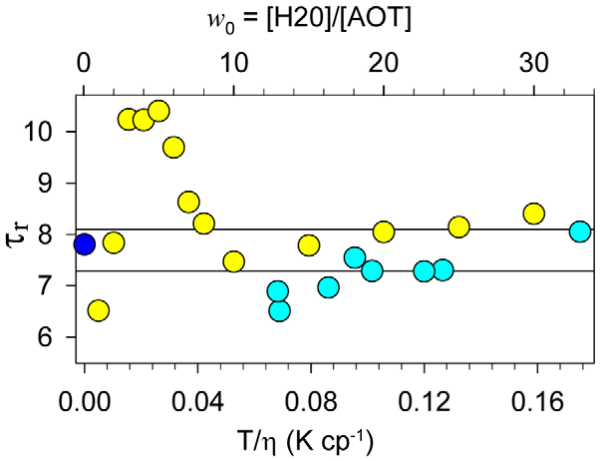
Radiative lifetime determined for thioflavin T in solutions of different viscosity and temperature (see **Table 1**) (open circles, bottom axis) and for the dye in the reverse micelles (open squares, top axis). Radiative lifetime for the dye in the reverse micelles were determined on the basis of the data given in the work [33], where *w*_0_ = [H2O]/[AOT] is the water to surfactant molar ratio. Radiative lifetime for the dye in rigid solution (*T*/*η*→0) (closed circle) was determined on the basis of the experimental dependence of 1/*q* on *T*/*η* and the value of fluorescence lifetime determined previously [31].

The radiative lifetime, determined as an average value of the ratio of fluorescence quantum yield and fluorescence lifetime of the excited state of ThT in solutions with different viscosity and temperature 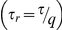, was estimated as *τ_r_* = 7.2 ns (Figure 4). The radiative lifetime for ThT in rigid environment was further evaluated based on the value of the fluorescence quantum yield at *T/η*→0 (*q_T_*_/*η*→0_ = 0.28, Figure 3, Insert) and on the value of the ThT excited state lifetime in rigid solutions of glycerol and n-propanol at *T* = 77K (*τ* = 2.2 ns) determined earlier [31]. Using these parameters, the radiation lifetime was determined to be *τ_r_* = 7.8 ns (Figure 4, closed circle). This evaluation is close to the radiative lifetime value obtained above (7.2 ns) and the value derived from the data obtained for the ThT in inverted AOT micelles with different ratios H_2_O and AOT (8.1 ns) [33]. In Figure 4, the data from [33] are presented in terms of *τ_r_* (open squares). Average *τ*_r_ value was determined based on the data corresponding to *w_0_* in the range from 30 to 10. The *w_0_* decrease from 10 to 5 leads to the dramatic increase in the *τ_r_* values. This is probably due to the specific interaction of AOT with the dye, which make the dye molecule more planar. The further decrease of *w_0_* leads to the *τ_r_* decrease practically to the values characteristic to the water solutions. Probably this means that ThT molecules do not incorporate into such micelles. Interestingly, the range of *w_0_* from 30 to 10 corresponds to the range of *T*/*η* values from 21 to 12 K·cp^−1^, whereas in experiments with water-glycerol mixtures, the *τ_r_* values were determined for ThT in solutions with *T*/*η* in the range from 0.18 to 0.07 K·cp^−1^ (Figure 4).

Extrapolation of the (1/ *q*−1) vs. *T*/*η* dependence gave a ThT fluorescent quantum yield in water at room temperature of *q*≈0.0001. Taking into account the determined radiative lifetime (7.8 ns), the lifetime of the ThT excited state under these conditions is shorter than 0.001 ns. Therefore, we can conclude that the data obtained based on the experiment with water-glycerol mixtures are in good agreement with the results obtained for the inverse micelles [33], [34].

### Temperature-dependence of the ThT fluorescence quantum yield

The main factor used in analysis of the dependence of ThT fluorescence quantum yield on temperature is the temperature dependence of the solvent viscosity, which is described by the equation [32]:
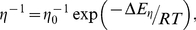
(5)where Δ*E_η_* is the activation energy of the solvent viscous flow. For water, this value is 3.7 kcal·mol^−1^ and data for water-glycerol mixtures with high content of glycerol are given in Table 2. These values were obtained on the basis of data from the study [31]. Combination of equations (4) and (5) resulted in the following equation:
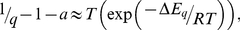
(6)where Δ*E_q_* is the activation energy of non-radiative deactivation of the excited state. Therefore, the experimental data were fitted to linear dependence in a 
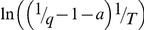
 vs 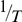 plot (Figure 3). The activation energy of the process of non-radiative deactivation of ThT determined based on the temperature dependence of fluorescence quantum yield Δ*E_q_* is slightly larger than the activation energy of the solvent viscous flow Δ*E_η_* (Table 2). A possible explanation for this phenomenon is the existence of some other factors (along with viscosity) preventing transition of the ThT molecule from its fluorescent to non-fluorescent state with the disturbed π-conjugated system of bonds. One of these factors is the electrostatic interaction of ThT molecule in the excited state with the molecules of the polar solvent.

**Table 2:** Comparison of activation energy of the solvent viscous flow (Δ*E_η_*) with the activation energy of the non-radiative deactivation of excited state (Δ*E_q_*) (determined by the temperature dependence of fluorescence quantum yield at high glycerol content in solution).

glycerol content, % wt	Δ*E_η_, kcal· mol^−1^*	Δ*E_q_, kcal· mol^−1^*
96.1	16.4	20.3
97.1	16.7	19.6
97.8	16.8	24.2
98.4	17.0	21.4
98.8	17.1	22.7

The ThT molecule has a positive charge (*Z* = +1e) that is non-uniformly distributed between the molecule fragments. Furthermore, this distribution depends on the angle *ϕ* between the molecule fragments and changes dramatically on molecule transition to the excited state [29]. The charged molecule with total charge *Z* can be represented as a molecule with a uniformly distributed charge *Z*/2 on each fragment and a dipole with the charge |*Z*_1_−*Z*_2_|/2. The larger the charge variation between the molecule fragments, the larger the dipole moment (|*Z*_1_−*Z*_2_|/2)×*r*, where *r* is the vector connecting geometrical centers of positive and negative charge. For the ThT molecule in the ground state, the charge allocation on the benzothiazole ring is +0.6, +0.7 and +0.8 and on the aminobenzyl ring, it is +0.4, +0.3 and +0.2. These three values correspond to the angle between the planes of the rings (*ϕ* equal to 0, 37 and 90°, respectively). For ThT in the excited state, the charge distribution is +0.5, +0.3 and −0.1 on the benzothiazole ring and +0.5, +0.7 and +1.1 on the aminobenzyl ring. This means that the transition to the excited state is accompanied by a reversal in the dipole direction. Therefore, in polar solution, the transition of the molecule to the excited state causes a state of significant non-equilibrium with the solvent. This non-equilibrium will decrease with *ϕ*→0 and increase with *ϕ*→90° and is equivalent to the existence of the energy barrier when ThT in the excited state transforms from the fluorescent to non-fluorescent state (*ϕ*→90°). Consequently, in the presence of polar solution, the transition of the molecule to the non-fluorescent state with ϕ close to 90° will be hindered in comparison to this transition for the isolated molecule.

### Fluorescence quantum yield of ThT incorporated into the insulin amyloid fibrils

**Figure 5:**
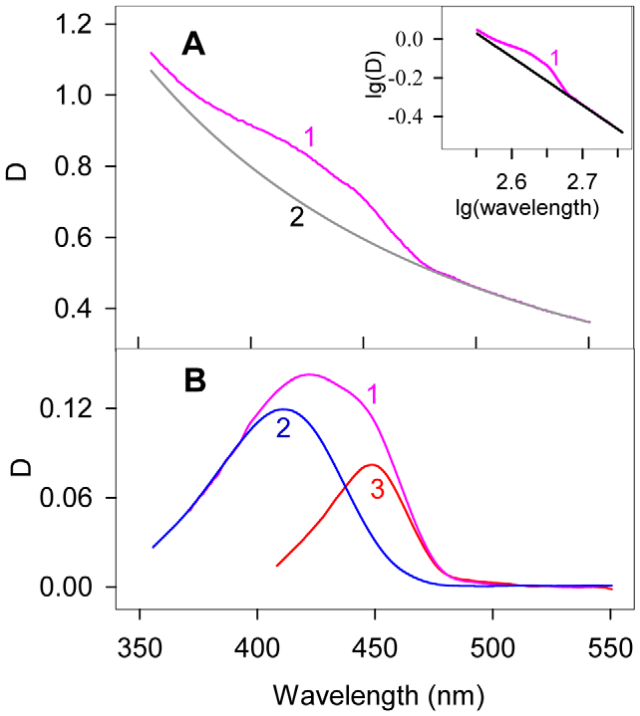
Absorption spectra of ThT incorporated into the insulin amyloid fibrils. Panel **a**. Curve 1 – Superposition of the absorption spectra of free ThT (at concentration *C_f_*), ThT bound to fibrils (at dye concentration *C_b_*) and the apparent absorption caused by fibrils light scattering; Curve 2 – Apparent absorption caused by fibrils light scattering. **Insert:** Determination of the apparent absorption caused by fibrils light scattering: *D_scat_ = aλ^−m^*. Coefficients *a* and *m* were determined from the linear part of the curve 1 plotted in logarithmic coordinates *lg(Dscat) = f(lg(λ))*. Panel **b**. Curve 1 is the total absorption of free and bound ThT after light scattering subtraction. Curve 2 is the absorption spectra of free ThT. Curve 3 is the absorption spectra of the ThT incorporated into the amyloid fibrils – the difference between 1 and 2 spectra. Insulin concentration was 6.9·10^−5^ M.

The fluorescence quantum yield of ThT bound to the amyloid fibrils, *q_b_*, was determined using the following equation:
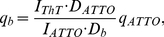
(7)where *I_ThT_* is the ThT fluorescence in the solution containing amyloid fibrils, *I_ATTO_* is the ATTO-425 fluorescence intensity in the solution with the optical density determined at the excitation wavelength to be equal to *D_ATTO_* = *D_b_+D_f_*, (where *D_b_* and *D_f_* are the optical densities of the bound and free ThT in solution containing amyloid fibrils). The quantum yield of the fibril-bound dye was measured in the solutions of amyloid fibrils and ThT prepared by the equilibrium dialysis. *D_b_* and *D_f_* values were determined from the absorption spectra of free and fibril-bound dye as described in legend to Figure 5. The fluorescence quantum yield of ThT incorporated into the amyloid fibrils was equal to 0.43. The noticeably higher values of the fluorescence quantum yield of ThT incorporated into the amyloid fibrils in comparison with the corresponding values measured for the dye in the rigid isotropic solution can be explained by the model where the ThT configuration became more planar at the dye embedding into the fibrils.

### Conclusions

Earlier [31], the dependence of ThT fluorescence quantum yield on solvent viscosity was explained assuming the existence of the unique way of the ThT excited state deactivation, namely the existence of the ultrafast intramolecular twisting of the dye fragments relative to each other. According to this model, the ThT fluorescence quantum yield in rigid solution was expected to be equal to unity. In this study, we used the fluorescence dye ATTO-425 with spectral characteristics close to that of ThT as a reference. This permitted the determination of the ThT fluorescence quantum yield in a wide range of viscosity and temperature. The study demonstrated that the dependence (1/*q*−1) on *T/η* is linear and extrapolation of this dependence allowed determination of the fluorescence quantum yield in water at room temperature (*q*≈0.0001) and showed that ThT fluorescence quantum yield in rigid solution is lower than unity: *q_T/η_*_→0_ = 0.28. This means that along with non-radiative deactivation of excited state by torsional oscillations of the ThT fragments relative to each other, leading to a twisted conformation (*ϕ* = 90°), there is some other cause of deactivation. We propose that this deactivation is due to the non-planar conformation of the ThT molecule in the ground state and consequently, in the Franck-Condon excited states. Determination of the fluorescence quantum yield and the lifetime of the excited state (*τ*) for ThT in solutions of different viscosity and temperature allowed the determination of the radiative lifetime (*τ*_r_ = 7.8 ns) and the calculation of excited state lifetime for ThT in water at room temperature (*τ*≈0.001 ns). Our results suggested that the fluorescence quantum yield of ThT incorporated into amyloid fibrils is determined not only by the steric restrictions of the ultra-fast twisting of the ThT fragments relative to each other, but also by the conformation of the bound ThT molecule.

It is evident now that fibrils formed by different proteins, or even by the same protein but at different conditions, are not structurally identical [35]. This potentially might result in the noticeable differences in the bound ThT fluorescence intensities. Furthermore, even in a single amyloid fibril, there could be several geometrically different binding sites [36], [37], [38] that are able to interact with different conformations of the dye and therefore resulting in differently bound ThT molecules having dissimilar fluorescence quantum yields. We showed that the ThT bound to the insulin amyloid fibrils is characterized by the fluorescence quantum yield which was noticeably higher than that measured in the rigid isotropic solution (q = 0.43 vs. q = 0.28). We believe that this difference is determined by the conformational difference between the dye molecule in the fibril-bound form and in the rigid isotropic solution, where the fibril-bound ThT is characterized by more planar structure. Consequently, the ThT fluorescence quantum yield can be used to characterize the peculiarities of the fibrillar structure. These observations clearly open new perspectives for the utilization of ThT in structural characterization of amyloid fibrils.

## Materials and Methods

### Materials

ThT from Sigma-Aldrich (USA) and Fluka (Switzerland) was purified by crystallization from 3∶1 (v/v) acetonitrile with ethanol [27]. ThT “Ultra Pure Grade” from AnaSpec, (USA), glycerol from Merck, (Germany) and fluorescence dye ATTO-425 from ATTO-TEC, (Germany) were used without further purification.

The samples of insulin and buffer components from Sigma (USA) were used without additional purification. Insulin amyloid fibrils were generated according to the standard protocol described earlier [39]. The concentration of insulin in the fibrillar form was determined based on the concentrations of the protein prior to fibrillation. Viscosity of the water-glycerol mixtures was estimated on the basis of glycerol concentrations determined by Abbe refractometer, LOMO, (Russia) at 23°C. The temperature dependence of the viscosity of different water-glycerol mixtures was taken from the literature [40]. ThT concentration in solutions was 1.4×10^−5^M.

### Steady-state and time-resolved fluorescence

Measurements were taken using the spectrofluorometers described earlier [41]. Absorption spectra were analyzed using a EPS-3T (Hitachi, Japan) spectrophotometer.

### Fluorescence quantum yield determination

**Figure 6:**
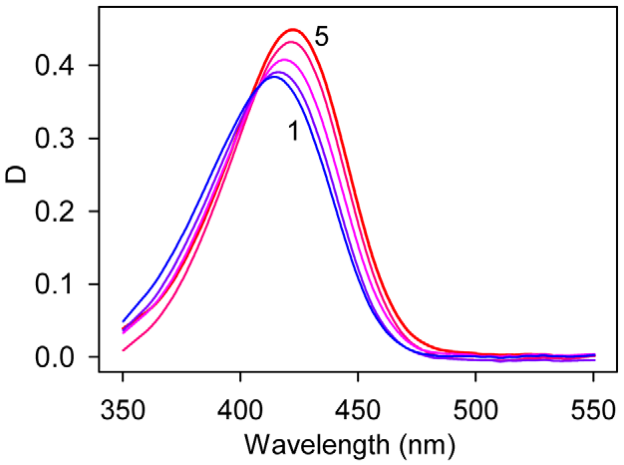
Absorption spectra of thioflavin T in water-glycerol mixtures. Curves 1–5 correspond to 13, 35, 56, 83 and 99% wt glycerol content, respectively.

When determining fluorescence quantum yield, the dependence of the ThT molar extinction coefficient on the content of glycerol in the water-glycerol solution was taken into account (Figure 6). The recorded fluorescence intensity was corrected on a solvent refractive index [42]. The independence of the ThT fluorescence spectrum from glycerol content in water-glycerol mixtures suggested that the fluorescence quantum yield is proportional to the fluorescence intensity (λ_em_ = 480 nm). An aqueous solution of fluorescent dye ATTO-425 with known quantum yield (*q* = 0.9) was used as a reference. Fluorescence was excited at 435 nm and recorded at 480 nm.

### Analysis of the fluorescence decay

Decay curves were fitted using the non-linear least-squares method. Minimization was accomplished according to Marquardt [43]. Experimental data were analyzed using the multiexponential approach:
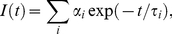
(8)where *α_i_* and *τ_i_* are amplitude and lifetime of component *i*, 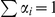. The root-mean square value of fluorescent lifetimes, 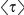, for biexponential decay is determined as:
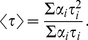
(9)

